# When One Hemisphere Takes Control: Metacontrol in Pigeons (*Columba livia*)

**DOI:** 10.1371/journal.pone.0005307

**Published:** 2009-04-23

**Authors:** Ruth Adam, Onur Güntürkün

**Affiliations:** 1 Department of Biopsychology, Institute of Cognitive Neuroscience, Faculty of Psychology, Ruhr-University Bochum, Bochum, Germany; 2 International Graduate School of Neuroscience, Ruhr-University Bochum, Bochum, Germany; University of Southern California, United States of America

## Abstract

**Background:**

Vertebrate brains are composed of two hemispheres that receive input, compute, and interact to form a unified response. How the partially different processes of both hemispheres are integrated to create a single output is largely unknown. In some cases one hemisphere takes charge of the response selection – a process known as metacontrol. Thus far, this phenomenon has only been shown in a handful of studies with primates, mostly conducted in humans. Metacontrol, however, is even more relevant for animals like birds with laterally placed eyes and complete chiasmatic decussation since visual input to the hemispheres is largely different.

**Methodology/Principal Findings:**

Homing pigeons (*Columba livia*) were trained with a color discrimination task. Each hemisphere was trained with a different color pair and therefore had a different experience. Subsequently, the pigeons were binocularly examined with two additional stimuli that combined the positive color of one hemisphere with a negative color that had been shown to the other, omitting the availability of a coherent solution and confronting the pigeons with a conflicting situation. Some of the pigeons responded to both stimuli, indicating that none of the hemispheres dominated the overall preference. Some birds, however, responded primarily to one of the conflicting stimuli, showing that they based their choice on the left- or right-monocularly learned color pair, indicating hemispheric metacontrol.

**Conclusions/Significance:**

We could demonstrate for the first time that metacontrol is a widespread phenomenon that also exists in birds, and thus in principle requires no corpus callosum. Our results are closely similar to those in humans: monocular performance was higher than binocular one and animals displayed different modes of hemispheric dominance. Thus, metacontrol is a dynamic and widely distributed process that possibly constitutes a requirement for all animals with a bipartite brain to confront the problem of choosing between two hemisphere-bound behavioral options.

## Introduction

**Figure 1:**
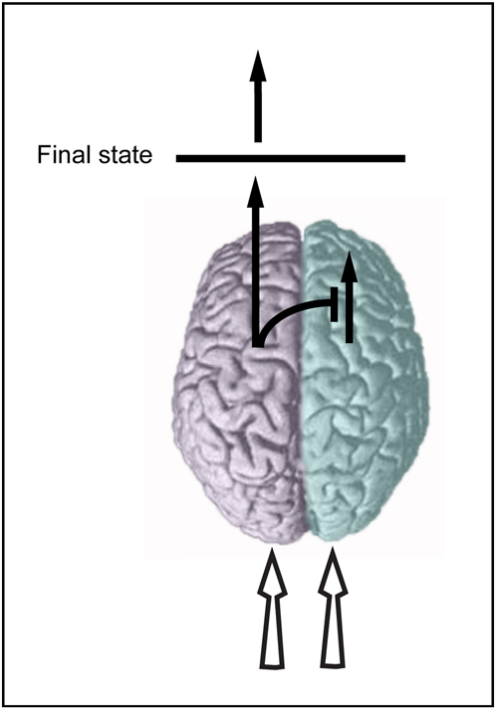
Schematic description of metacontrol. In some cases, a task that is viewed by both hemispheres, i.e. bilateral input, is dominated by a single hemisphere. Metacontrol is the mechanism that determines which hemisphere it will be. The phenomenon was proposed to occur by means of an inhibitory influence exerted by the behaviorally dominant hemisphere upon the other hemisphere.

Since the pioneering study of Broca in the nineteenth century, it is widely known that each of the two cerebral hemispheres processes and computes information differently. As outlined below, several studies report that this asymmetrical organization can be accompanied by unilateral control over a task. In this case, the performances under bilateral viewing are similar to the performances of a single hemisphere, and different from the performances of the other half-brain. The choice mechanism that determines which hemisphere will dominate the task is known as metacontrol [1]. The term does not infer that the non-dominating half brain is not involved at all but specifies that the observed behavior is primarily guided by the metacontrolling hemisphere. The occurrence of metacontrol can be explained by computational costs. Given a lateralized brain, it is more beneficial to process simple tasks using one hemisphere than to invest in time- and energy-consuming integration [2]. Metacontrol will then occur possibly by inhibition of the other hemisphere [3] (Figure 1).

Both left hemisphere (LH) metacontrol, meaning left hemisphere control over the task, and right hemisphere (RH) metacontrol were observed in humans [4], [5], [6]. Surprisingly however, the dominating hemisphere is not always the specialized one [1]. For example, in a verbal task the binocular performances were identical to those under RH viewing, although unilaterally the left hemisphere performed the verbal task better than the right hemisphere [6]. Which hemisphere will take control is a widely unexplored question, but it is known that task specifications affect hemispheric dominance. Known properties that shape hemispheric dominance are hemispheric stimulation timing [7], task's instructions [1], and the input-processing strategy [8]. Thus, metacontrol could result from an interhemispheric winner-takes-all mechanism in which a small advantage of one hemisphere produces unilateral dominance during the task via commissural inhibition.

Metacontrol has been shown in healthy humans [4]–[8] as well as in split brain patients [1]. For example, Hellige et al., 1988 [4] asked normal human subjects to judge whether two faces, which could vary along four features, are similar or different. One of the faces was presented bilaterally and the other could be presented either to the left hemisphere (right visual field), to the right hemisphere (left visual field), or to both hemispheres (bilateral presentation). By analyzing how the reaction time and the accuracy of the decision were affected by the specific incompatible features, the authors inferred the mode of processing that occurred. In most of the subjects, varying a specific facial feature between the two faces similarly affected their reaction times under left hemisphere and bilateral presentations. In other words, the performances by the left hemisphere were similar to those under bilateral presentation, and were significantly different from those by the right hemisphere. This pattern reflects LH-metacontrol. A few subjects showed the opposite relation, demonstrating RH-metacontrol, and in a few others the bilateral performances were similar to the average of both unilateral presentations, implying shared computation by both hemispheres.

To our knowledge, apart from humans, metacontrol was examined only in monkeys [9]. In this one study, two split brain macaques had to decide whether a stimulus had been shown before or was novel. During test trials both hemispheres each received visual input that were either identical or different. The two monkeys employed two different strategies: one monkey utilized LH-metacontrol and the other showed equal contribution of both hemispheres.

Both humans and macaques have frontally placed eyes that have a single fovea. Foveated objects are perceived by both hemispheres. Since primates produce a very high amount of eye movements, both half-brains see the majority of objects in the front of the animal. Conflicting and response-demanding input into the hemispheres is therefore not a major problem as long as stimuli are foveated. This is radically different for most birds. All birds have a virtually complete crossing of their optic nerves, transmitting visual input to the contralateral hemisphere [10]. Most birds also have laterally placed eyes with only a small binocular overlap [11]. Birds scrutinize objects mostly with their lateral monocular visual field before deciding to approach and peck [12]. Thus, response selection is mostly performed under conditions of unilateral visual input. The aim of the current study was to see whether metacontrol occurs in birds. To this end, we tested homing pigeons (*Columba livia*) in a simple color discrimination task that these birds master quickly, and for which there are little or no hemispheric differences [13], [14]. The pigeons were trained monocularly to discriminate a different color pair with each hemisphere. Binocularly they were then tested with stimuli that combined a positive color according to one hemisphere with a negative color according to the other, resulting a conflicting situation. Under conditions of such hemispheric stalemate we indeed observed metacontrol in pigeons.

## Methods

### 1. Subjects

14 pigeons were the subjects of this study. Five were naïve and the rest had participated in former, unrelated, experiments. The birds were housed individually in a room with other conspecifics and placed on a 12/12h light/dark cycle. They were kept at 80–90% of their free feeding weight. Food was provided during the experiment and after experimental sessions. Water was freely available in their home cages throughout the experimental period. The pigeons were trained on average 6 times a week.

### 2. Ethics Statement

The experiment was conducted according to the specifications of the German law for the prevention of cruelty to animals and hence, the European Communities Council Directive of 24 November 1986.

### 3. Apparatus

The experiment was conducted in a 33(w)×34.5(d)×36(h) cm custom made Skinner box. The box was equipped with a house light on the side panel, a centered feeder containing mixed grains (on the front panel, 14 cm from the ceiling, 5 cm from the right side), a feeder light located above the feeder that was lit simultaneously with the feeder activation. Additionally, a centrally located transparent pecking key was located on the front panel, with its upper right corner being located 14(w)×7.5(h) cm from the upper right corner of the Skinner box. Through the pecking key, the pigeons viewed the 5(w)×2.8(h) cm stimuli that were presented on a TFT LCD monitor (Brilliance 150P_2_, Philips), with a resolution of 1024×768 Pixels. Pecking correctly on the pecking key reinforced the pigeons with the activation of the feeder. Experimental sessions and data collection were controlled by a Pentium PC running MATLAB (The MathWorks, Inc., Natick, MA, USA) and a partial pre-version of Biopsy Toolbox [15].

### 4. Stimuli

**Figure 2:**
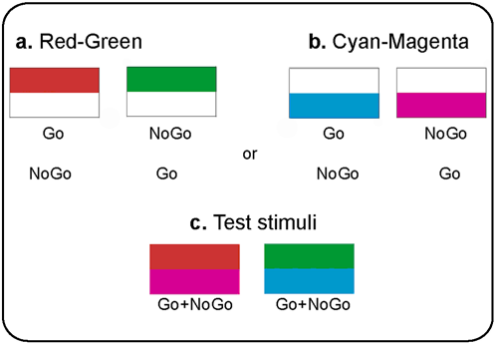
Experimental stimuli. Stimuli used for the monocular discrimination training: (a.) Red-Green stimuli (b.) Cyan-Magenta stimuli (c.) The conflicting test stimuli that were presented to the pigeons binocularly. Those stimuli were a combination of a Go color according to one hemisphere, together with a NoGo color according to the other hemisphere.

The stimuli used were 5(w)×2.8(h) cm rectangles. Training stimuli were half colored: the Red and Green stimuli were colored in their upper half, while the Cyan and Magenta stimuli were colored in their lower half (Figure 2a). For seven pigeons, Red and Cyan were the positive colors (Go) and Green and Magenta were the negative colors (NoGo), and vice versa for the other seven pigeons. Stimuli were learned monocularly.

Test stimuli were a combination of a Go training color with a NoGo belonging to the other color pair, i.e. Red-Magenta or Green-Cyan (Figure 2b). The test stimuli were viewed binocularly. All stimuli were viewed through the centrally located pecking key.

### 5. Procedure

#### Initial training

The five naïve pigeons were autoshaped to peck on a lighted pecking key (white square) in a standard autoshaping procedure containing 40 trials. The white square was presented for 5 seconds followed by 3 seconds of food access. After the pigeons started to respond to the pecking key, they were trained with a continuous reinforcement schedule. Subsequently, the pigeons were progressively trained with variable ratio (VR4, VR7, VR10), fixed interval (FI3, FI5, FI10) and variable interval (VI10, VI15 and VI20) schedules. Each schedule proceeded until the pigeons responded correctly to more than 85% of the trials in two consecutive sessions. Each session contained 40 trials. Afterwards, they were monocularly trained in a VI20 schedule, in order to make them familiar with wearing and working with an eye cap. The other nine pigeons were already familiar with the Skinner box and the eye caps.

#### Monocular discrimination training

Monocular viewing was made possible using eye caps. A velcro ring was fixed to the skin around the eyes using non-toxic glue. A cap could be attached to the ring, blocking the view of this eye and thus the contralateral hemisphere. The pigeons were adapted to the caps prior to the monocular testing sessions by wearing them in their home cages. The animals wore a cap for about 25 minutes before each testing session.

A Go-NoGo task was used to teach the pigeons the discrimination. The schedule used was similar to the one used by Yamazaki et al. [16]. A trial began with 20 s inter-trial interval. Next, a stimulus was presented for 10 s FI and then for 5 s VI. In a Go (positive) trial the pigeons had to respond two or more times by pecking on the pecking key, and were subsequently rewarded with 3 s food access, accompanied by an illumination of the feeder. In a NoGo (negative) trial, a stimulus was presented for additional 8 s after the VI period, in which the subjects had to refrain from responding. A NoGo trial was terminated only after no response occurred for 8 s. Each session consisted of 40 trials that appeared pseudo-randomly so that no more than 3 Go or NoGo trails appeared consecutively. On average, half of the trails were Go trails.

As the pigeon optic nerve decussates virtually completely at the optic crossing, each hemisphere can be tested separately by occluding one eye [10], [11]. The pigeons were trained monocularly in a color discrimination task. Each hemisphere was trained to discriminate a different color pair. The pigeons were divided into four groups, which differed in terms of the stimuli pair each hemisphere was trained with as well as their contingencies:

1. Four pigeons were trained in a Red/Green (Go/NoGo) color discrimination with the left hemisphere and a Cyan/Magenta discrimination with the right hemisphere.
2. Four pigeons were trained in a Green/Red discrimination with the LH and a Magenta/Cyan discrimination with the RH.
3. Three pigeons were trained in a Cyan/Magenta discrimination with the LH and a Red/Green discrimination with the RH.
4. Three pigeons were trained in a Magenta/Cyan discrimination with the LH and a Green/Red discrimination with the RH.

Each of the two hemispheres was tested alternately.

The discrimination criterion was rho≥.9 in two out of three consecutive sessions, for both hemispheres.

#### Test session

The test stimuli were either Go-color learned by the left hemisphere combined with a NoGo-color trained by the right hemisphere (LH-Go & RH-NoGo), or a Go-color trained by the right hemisphere combined with a NoGo-color trained by the left hemisphere (LH-NoGo & RH-Go).

The binocularly seeing test sessions contained six stimuli: the four monocularly-learned stimuli: LH-Go (the Go-color learned by the LH), LH-NoGo (the NoGo-color learned by the LH), RH-Go and RH-NoGo, as well as the two critical test stimuli: LH-Go combined with RH-NoGo and RH-Go combined with LH-NoGo ((LH-Go & RH-NoGo) and (LH-NoGo & RH-Go), respectively). Each of the six stimuli appeared 8 times. The stimuli were presented in a random order that was changed among the pigeons. Test stimuli were not reinforced.

### 6. Analysis and Statistic

The rho value was used to index performances [17]. Rho compares the number of pecks in Go versus NoGo trials in a single session using the U value of the Mann-Whitney U test divided by the product of the number of Go and NoGo trials.

The *Laterality index* indicated if binocularly there was a performance difference between the LH-learned and the RH-learned color information. It was measured using the rho values obtained from the binocular discrimination of the monocularly–learned color pairs.

The laterality index was calculated by the following formula:
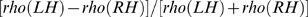
Where laterality index = 1 indicated a total discrimination of the LH-learned color pair, and a complete lack of discrimination of the RH-learned color pair.

The *Dominance index* indicated the type of hemispheric interaction during the conflicting situation. The dominance index was computed by the following formula, using the rho values calculated from the performances with the test stimuli:
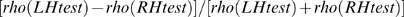

*rho(LHtest)* is the rho value for the number of times the pigeon pecked on the test stimulus containing the Go color learned by the LH: (LH-Go & RH-NoGo) relative to the number of pecks on the other test stimulus containing the Go color learned by the RH: (RH-Go & LH-NoGo).

*rho(RHtest)* is the rho value for the number of times the pigeon pecked on the test stimulus containing the Go color learned by the RH (RH-Go & LH-NoGo) relative to the number of pecks on the test stimulus containing the Go color learned by the LH (LH-Go & RH-NoGo).

Bootstrap analysis was further performed to determine the likelihood of receiving the obtained dominance index values. The analysis was done for every animal separately by randomly assigning the pecks in the 16 test trials into Go and NoGo groups, for 1000 times. Following the reassignment, the distribution of dominance index value was computed, and a Z-score was used to calculate the probability of the obtained index.

One sample t-test was used to calculate if the laterality index and the dominance index differ from zero. Using paired t-tests we compared the performances of the two hemispheres. A 2×2 repeated measures ANOVA with the factors session (last monocular session vs. binocular session) and Hemisphere (RH vs. LH) analyzed the performances with the monocularly-learned stimuli. Pearson correlations were further used.

Means values are reported in the format of mean±SEM.

## Results

### Monocular discrimination training

The discrimination criterion was attained when performances reached rho≥.9 in two out of three consecutive sessions, for both the two hemispheres consecutively. On average, the pigeons needed 10.9±1.8 sessions (ranged from 5 to 28) to reach rho≥.9 with the left hemisphere, and 8.7±1.8 sessions (ranged from 3 to 27) with the right hemisphere. Nine pigeons achieved high performance more quickly with their right hemisphere, four with the left hemisphere, and one pigeon needed equal numbers of sessions with both hemispheres. This difference in acquisition speed was not significant (t(13) = −0.944, *p* = .362). Since reaching the discrimination criterion and moving to the test session depended on both hemispheres, in some pigeons the hemispheres were overtrained. Thus, overall, with the left hemisphere the pigeons were trained on average for 13.1±2.6 sessions, and had 13±2.6 sessions with the right hemisphere. The average performances in the last training session were rho = .971±.005 and rho = .975±.007 with the left- and the right-hemisphere, respectively. The hemispheres did not differ in their performances in the last training session (t(13) = −.436, *p* = .670).

### Test session

During the binocular test session the pigeons were confronted with six stimuli: the four stimuli known from the monocular training as well as the two conflict-producing stimuli that produced a Go-response in one and a NoGo-response in the other hemisphere.

**Figure 3:**
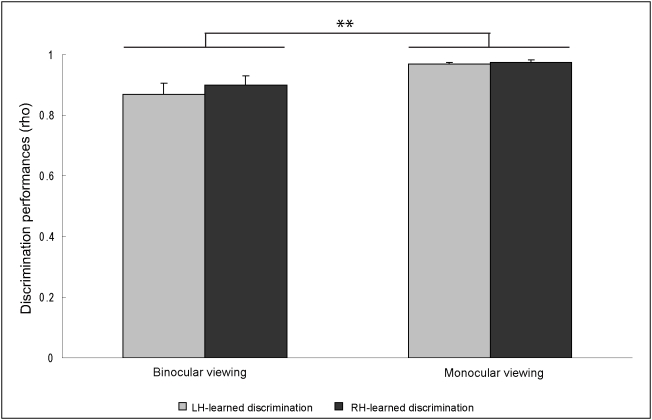
Comparison of monocular versus the binocular discrimination performances. No difference in discrimination accuracy between the two hemispheres was detected. However, the binocular performances were significantly worse than the monocular ones (*p* = .001).

The binocular performances with the monocularly-learned stimuli were rho = .87±.036 (range: from rho = .523 to rho = 1) with the color pair learned by the left hemisphere, and rho = .90±.03 (range: from rho = .625 to rho = 1) with the color pair learned by the right hemisphere (Figure 3). The laterality index did not differ significantly from zero (average = −.018±.033, t(13) = −.537, *p* = .600). Interestingly, the pigeons performed the color discrimination task better in the last monocular viewing session compared with the binocular viewing session (F(1,13) = 18.471, *p* = .001; pairwise comparison monocular vs. binocular performances: .088±.021, *p* = .001 Bonferroni corrected) (Figure 3). The performances were independent of which hemisphere learned the tasks (hemisphere main effect: F(1,13) = .369, *p* = .554, interaction: F(1,13) = .196, *p* = .665).

**Figure 4:**
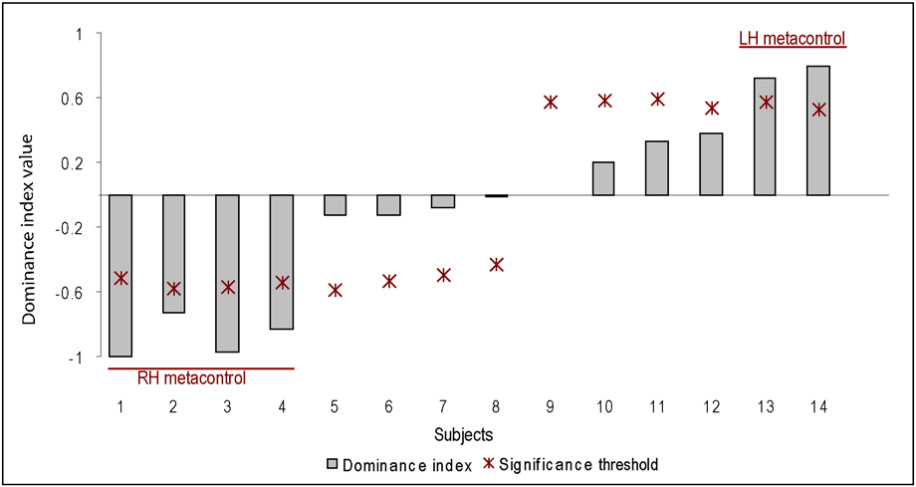
The distribution of hemispheric dominance. Gray bars show the dominance index found in the experiment for every pigeon. Red stars mark significant threshold according to the bootstrap. Four pigeons showed RH dominance, or RH-metacontrol, two exhibited LH-metacontrol and in the other eight pigeons both hemispheres contributed similarly to the task.

Seeing the test stimuli, i.e. (LH-Go & RH-NoGo) and (LH-NoGo & RH-Go), the pigeons were faced with a conflicting situation. For every test trial, the pigeons had to decide according to which monocularly-learned color pair, i.e. hemisphere, they would react. Hemispheric dominance was determined by the pigeons' relative choices with the two test stimuli. The pigeons showed complete distribution of hemispheric dominance, ranging from .8 to −1. Across the group, the hemispheric dominance was normally distributed (mean dominance index = −.104±.157, t(13) = −.661, *p* = .520). Nonetheless, comparing the average bootstrap index of each pigeons to its measured index, as well as looking on the U value from which the rho value was obtained, showed that six pigeons exhibited significant metacontrol. Four pigeons had a significantly negative dominance index, and hence showed RH-metacontrol, whereas two pigeons showed LH-metacontrol as their dominance index was significantly positive (Figure 4).

The degree of metacontrol was correlated with the binocular discrimination of the LH-trained but not the RH-trained stimuli (LH: r (14) = .710, *p* = .004; RH: r(14) = −.438, *p* = .117). Finally, the dominance index was approaching a significant correlation with number of monocular training sessions needed by the left hemisphere till criterion (LH: r(14) = .522, *p* = .055; RH: r(14) = .207, *p* = .472). The dominance index, however, was not correlated with the amount of overtraining sessions that occurred while a hemisphere was waiting for the other to reach criterion (LH: r(14) = .036, *p* = .904; RH: r(14) = . 389 , *p* = .170).

## Discussion

Metacontrol refers to the existence of a preference or choice mechanism that determines which hemisphere will control a task when the two sides of the brain are facing discrepant behavioral options [7]. This phenomenon had only been demonstrated in humans [1], [4], [8] and in split brain monkeys [9]. Due to their laterally placed eyes metacontrol should even be more likely in the majority of bird species. Indeed, we could show that pigeons display metacontrol with characteristics similar to the ones known from humans. Thus, the ability to switch dominance to a single hemisphere in moments of conflict seems to be an ancient mechanism of the vertebrate brain.

In the initial training the pigeon were taught to discriminate between two color pairs, one with each hemisphere. This is an easy task for pigeons, and as shown previously [13], we found no difference in acquisition time between the two hemispheres, possibly due to a ceiling effect. Interestingly, the pigeons discriminated the monocularly-learned colors better under monocular conditions than under binocular viewing. Thus, although these stimuli were non-contradictory, bilateral exposure possibly invoked the participation of both hemispheres of which only one had been previously trained, thus creating interference between them. Similarly, when human subjects have to judge the congruence of two faces, their reactions are slower and less accurate when target faces appear bilaterally compared to unilateral stimulation [4]. Similar findings in humans also show the relevance of task requirements. Dividing a simple comparison task between the two hemispheres impairs performance, whereas subjects benefit from resource sharing of the two hemispheres when faced with a more demanding undertaking [18]. The color discrimination, as used in our study, is learned extremely fast by pigeons and thus constitutes a simple task. Thus, in such a case, a monocular advantage could follow. Under conditions in which unilateral control is superior to the bilateral one, unilateral dominance of the final behavioral output should be advantageous and indeed it occurred.

As previously found in humans [4], [8], [19] and monkeys [9], the pigeons showed variation in the metacontrol distribution. Four pigeons evinced RH-metacontrol, and the behavior of two other pigeons was dominated by the left hemisphere. Our results hint of a mechanism that determines which hemisphere will control the task. The more sessions the left hemisphere required, i.e. the more exposure it had to the stimuli, the more LH-dominance was observed. Interestingly, this was not the case for the right hemisphere, and the four pigeons that showed RH-metacontrol required little training with the right hemisphere till criterion. Although the correlation between the number of sessions required by the left hemisphere and the dominance degree only approached significance, we believe that it has important meaning. Similarly, in humans, the timing of hemispheric stimulation, modulated by the initial hemispheric dominance in the task, was suggested as one of the factors that affects hemispheric dominance in bilateral stimulation [7]. Together these data suggest that hemispheric specialization and hemispheric exposure both affect metacontrol.

The half brain that has a slight advantage, either previously or due to training, seems able to take control over the task, possibly via commissural inhibition. In mammals this could be achieved with the corpus callosum. Since birds do not possess this commissure, other interhemispheric inhibitory pathways at brainstem level are obviously also able to achieve a similar function. Indeed, the intertectal commissures in birds are mostly inhibitory [20], [21]. Additionally, the bilateral integration of the ascending streams of the tectofugal system is selectively inhibited by GABAergic fibers from a cluster of nuclei, collectively called bed nuclei of the tecto-thalamic tract [22]. Taken together, metacontrol is not necessarily related to the corpus callosum, but can possibly be established with subcortical inhibitory commissural systems.

The lines to today's birds and mammals parted about 280 million years ago [23]. The sharing of mechanisms leading to metacontrol in pigeons, macaques, and humans might indicate a long and common history of this neurocognitive mechanism. In fact, we assume that the neural mechanisms for metacontrol could even date farther back since the problem to create a singular behavioral output from a bipartite brain should be shared by all vertebrates.
